# A tale of two yeasts: *Saccharomyces cerevisiae* as a therapeutic against candidiasis

**DOI:** 10.1080/21505594.2016.1230580

**Published:** 2016-08-31

**Authors:** Duncan Wilson

**Affiliations:** Aberdeen Fungal Group, MRC Center for Medical Mycology, School of Medicine, Medical Sciences and Nutrition, University of Aberdeen, Institute of Medical Sciences, Aberdeen, UK

**Keywords:** *Candida albicans*, *Saccharomyces cerevisiae*, vulvovaginal candidiasis

Normally a benign commensal colonizer of mucosal surfaces such as the gastrointestinal tract, *Candida albicans* is also one of the most common fungal pathogens of humans, responsible for both superficial as well as life-threatening invasive infections. Arguably the commonest type of infection caused by *C. albicans* is vulvovaginal candidiasis (VVC), as it affects 75% of women of childbearing age.^1^ And unlike many other manifestations of candidiasis, which are associated with particular immune deficiencies, VVC frequently occurs in otherwise healthy women.

Symptomatic VVC can be associated with the use of (antibacterial) antibiotics. It is thought that elimination of the protective bacterial vaginal flora allows *Candida* overgrowth and symptomatic disease. Indeed, for diseases of normally non-sterile sites such as mucosae, the balance of the microbial community (the microbiota) has a very important impact on the outcome of a particular host-pathogen interaction, and the presence of commensal organisms can elicit “colonization resistance” against potential pathogens.^2,3^ In this context, the principle of preventing or treating microbial infections with other microbes, has a long history, and a number of studies suggest that such approaches can be clinically beneficial. For example, the commensal species *Clostridium scindens* can protect mice from *Clostridium difficile* infections.^4^ Because *Lactobacilli* species are the dominant microbiota of the healthy vagina, imbalances in these species are implicated in VVC and there is some evidence for protective effects of *Lactobacillus* probiotic administration.^5^

In this issue of *Virulence*, Pericolini and colleagues looked at the effect of *Saccharomyces cerevisiae* yeast administration on the course of infection in a murine model of VVC. They found that postinfection vaginal administration of live or inactivated *S. cerevisiae* enhanced clearance of infecting *C. albicans* cells.^6^ The authors used *in vivo* imaging of mice infected with a *C. albicans* strain expressing the luciferase bioluminescent protein. Using this technique, it is possible to noninvasively track, over time, the progression, and resolution of VVC in living mice. Intriguingly, they found that a single administration of live *S. cerevisiae* cells elicited *Candida* clearance at levels comparable to treatment with the commonly used antifungal drug, fluconazole. Of note, even inactivated yeast cells elicited fungal clearance, but this was not as sustained as was observed for viable *Saccharomyces* cells. This suggested that *S. cerevisiae* cells may be used therapeutically for the treatment of VVC; but how do yeast cells help to resolve VVC?

*C. albicans* infections of mucosal surfaces rely on a complex number of interlinked fungal pathogenicity mechanisms and virulence factors centered around the fulcrum of hyphal morphogenesis.^7^ Primarily, the pathogen must adhere robustly to its host to initiate colonization, and hypha formation strengthens adhesion potential substantially due to the expression of hypha co-expressed genes which encode the adhesin proteins Als3 and Hwp1, among others.^8^ *C. albicans* hyphae are also essential for epithelial invasion, not only due to the penetrative nature of the extending filament, but because the hypha coexpressed adhesin Als3 also functions as an invasin, triggering fungal uptake by epithelial cells.^9^ Although this process has not yet been tested for *C. albicans*-vaginal cell interactions, induced endocytosis via Als3-cadherin interactions is an established mechanism of fungal invasion of both oral epithelial and endothelia cells.^10^

*C. albicans* also produces an array of factors which can damage host tissue at mucosal surfaces, including secreted hydrolases such as aspartyl proteases, lipases and phospholipases and a cytolytic toxin called Candidalysin.^11,12^ In combination, the activities of hypha formation, adhesion, invasion, as well as hydrolase and cytolysin secretion can damage epithelial cells and trigger epithelial immunity.^13^ However, in the context of vaginal candidiasis, proinflammatory responses seem to do more harm than good, as symptomatic disease appears to be the result of an aggressive, and nonprotective, influx of neutrophils.^14^

Pericolini *et al.* therefore questioned whether the therapeutic effect of *Saccharomyces* cells observed in their animal infection model of VVC was due to interference with or modulation of specific *C. albicans* pathogenicity factors. Using tissue culture models of *C. albicans*-epithelial interactions, they found that pre-treating the epithelia with either live or inactivated yeast cells inhibited *C. albicans* adhesion to the vaginal epithelial cells. This inhibition of *C. albicans* adhesion by both living and inactivated yeast cells was likely due to physical competition and aggregation between the fungi.

The authors next investigated the effect of *Saccharomyces* on *C. albicans* morphogenesis. Here, they found that living, but not inactivated yeast cells strongly repressed *C. albicans* hypha formation and even the cell free supernatant of *S. cerevisiae* cultures had an inhibitory effect. This is an important observation because the expression of multiple *C. albicans* pathogenicity- and virulence- factors is intimately linked to hyphal morphogenesis. That is, either chemical- or genetic- inhibition of the yeast to hypha transition also blocks *C. albicans* adhesion to, invasion and damage of mammalian epithelial cells.^8,15^

Similarly, viable *Saccharomyces* cells were also able to block the expression of *SAP2* and *SAP6* by *C. albicans* both *in vitro* and in the mouse model of VVC. These two genes encode secreted aspartyl proteases which have long been implicated in *C. albicans* virulence.^16^ Interestingly, Saps can also influence the inflammatory pathogenesis of VVC: administration of recombinant Sap2 to the vaginal cavity results in pronounced IL1β production and neutrophil influx in mouse models, even in the absence of infecting *Candida* cells.^17^ As such neutrophil influx appears to be nonprotective in the context of VVC^14^, and may actually drive the symptomatic pathogenesis of this disease, *Saccharomyces*-treatment might help resolve symptoms, not only by enhancing *C. albicans* clearance, but also by dampening inflammatory responses. In the future, it will be interesting to examine the impact of *Saccharomyces* administration on local inflammatory responses and neutrophil infiltration to the site of infection.

Therefore, viable *S. cerevisiae* cells not only physically perturb *C. albicans* colonization of epithelia, but also directly inhibit the elaboration of several key pathogenicity factors. This study underscores the complex interactions which can occur between microbial cells within a mammalian host and the impact these multi-species interactions have on the outcome of an infection.
